# An ultrastructural analysis of the effects of ethanol self-administration on the hypothalamic paraventricular nucleus in rhesus macaques

**DOI:** 10.3389/fncel.2015.00260

**Published:** 2015-07-14

**Authors:** Vanessa A. Jimenez, Christa M. Helms, Anda Cornea, Charles K. Meshul, Kathleen A. Grant

**Affiliations:** ^1^Behavioral Neuroscience, Oregon Health and Science UniversityPortland, OR, USA; ^2^Division of Neuroscience, Oregon National Primate Research CenterBeaverton, OR, USA; ^3^Research Services, Veterans Affairs Medical CenterPortland, OR, USA

**Keywords:** paraventricular hypothalamus, ethanol self-administration, hypothalamic-pituitary-adrenal axis, electron microscopy, corticotropin-releasing hormone, arginine-vasopressin, monkey

## Abstract

A bidirectional relationship between stress and ethanol exists whereby stressful events are comorbid with problematic ethanol use and prolonged ethanol exposure results in adaptations of the physiological stress response. Endocrine response to stress is initiated in the hypothalamic paraventricular nucleus (PVN) with the synthesis and release of corticotropin-releasing hormone (CRH) and arginine-vasopressin (AVP). Alterations in CRH and AVP following long-term ethanol exposure in rodents is well demonstrated, however little is known about the response to ethanol in primates or the mechanisms of adaptation. We hypothesized that long-term ethanol self-administration in nonhuman primates would lead to ultrastructural changes in the PVN underlying adaptation to chronic ethanol. Double-label immunogold electron microscopy (EM) was used to measure presynaptic gamma-aminobutyric acid (GABA) and glutamate density within synaptic terminals contacting CRH- and AVP-immunoreactive dendrites. Additionally, pituitary-adrenal hormones (ACTH, cortisol, DHEA-s and aldosterone) under two conditions (low and mild stress) were compared before and after self-administration. All hormones were elevated in response to the mild stressor independent of ethanol consumption. The presynaptic glutamate density in recurrent (i.e., intra-hypothalamic) CRH terminals was highly related to ethanol intake, and may be a permissive factor in increased drinking due to stress. Conversely, glutamate density within recurrent AVP terminals showed a trend-level increase following ethanol, but was not related to average daily consumption. Glutamate density in non-recurrent AVP terminals was related to aldosterone under the low stress condition while GABAergic density in this terminal population was related to water consumption. The results reveal distinct populations of presynaptic terminals whose glutamatergic or GABAergic density were uniquely related to water and ethanol consumption and circulating hormones.

## Introduction

Alcohol use disorders are a major public health concern. In 2014, 5.1% of the global burden of disease and injury was attributed to alcohol use [World Health Organization (WHO), 2014]. Stress is believed to be an etiological factor in the development of alcohol dependance (Keyes et al., 2012), and alterations in the stress response may contribute to continued alcohol consumption despite adverse consequences (Sinha, 2012). Hypothalamic-pituitary-adrenal (HPA) axis activation is integral to the definition of stress, which is defined as any stimulus, physical or psychological, that challenges homeostasis and activates the HPA axis (Smith and Vale, 2006). At the apex of the stress response are the parvocellular neurons of the hypothalamic paraventricular nucleus (PVN). In response to real (i.e., external or physical) or perceived (i.e., sensory or psychological) stressors, these neurons synthesize and release corticotropin-releasing hormone (CRH) and arginine-vasopressin (AVP). These neuropeptides induce HPA axis activation via CRH-R1 and AVP-1b receptors on corticotropes in the anterior pituitary, leading to synthesis and release of adrenocorticotropin hormone (ACTH). CRH has the greatest stimulatory effect on pituitary ACTH (Rivier and Vale, 1983a). AVP produces a mild ACTH-stimulatory effect on its own, but when co-released with CRH, ACTH release is greater than after either neuropeptide alone (Rivier and Vale, 1983b; Antoni, 1993). From circulation, ACTH initiates the synthesis and release of adrenal hormones in the zona fasciculata (cortisol), zona reticularis (dehydroepiandrosterone, DHEA; Conley et al., 2004; Nguyen and Conley, 2008) and zona glomerulosa (aldosterone; Williams and Williams, 2003). Cortisol binds to glucocorticoid receptors and acts as a negative feedback signal by reducing further activation of the HPA axis and restoring homeostasis. AVP, like CRH, is suppressed by glucocorticoid-induced negative feedback (Erkut et al., 1998). Activation of parvocellular neurons, as measured by c-Fos, depends on the type and magnitude of the stressor (Pacák and Palkovits, 2001) suggesting varied sources of synaptic input that code for stress intensity. The majority of synaptic contacts onto the parvocellular neurons of the PVN contain glutamate and gamma-aminobutyric acid (GABA; Decavel and van den Pol, 1992); although other neuropeptides and neurotransmitters are also present such as norepinephrine (Liposits et al., 1986; Daftary et al., 2000) and serotonin (Qi et al., 2009).

Acute ethanol produces an elevation in c-fos expression in parvocellular neurons and initiates secretion of the stress hormones ACTH and cortisol. However, repeated ethanol vapor exposure results in a blunted ACTH and cortisol response to self-administered and acute ethanol in rodents (Richardson et al., 2008). Ethanol’s ability to acutely increase pituitary ACTH, and its precursor preopiomelanocortin, is eliminated by CRH and AVP antibodies, indicating that ethanol impacts CRH and VP signaling in the hypothalamus (Lee et al., 2004). Hypothalamic parvocellular neurons have ionotropic N-methyl-D-aspartate glutamate receptors (Herman et al., 2000; Ziegler et al., 2005), GABA_A_ receptors (Cullinan, 2000) as well as alpha-1 adrenergic receptors (Day et al., 1999; Daftary et al., 2000), of which the former two are mechanisms for ethanol pharmacology (Grant and Lovinger, 1995). Both stress and ethanol are able to disrupt neurotransmission without necessarily having profound effects on receptor content or density. For example, Flak et al. (2009) studied biomarkers for noradrenergic, glutamatergic and GABAergic synaptic contacts following 1 week of chronic variable stress. The result was an increase in the number of excitatory synapses onto CRH-immunoreactive neurons in the PVN, likely contributing to hyper-excitability of the HPA axis, but no changes in GABA contacts. In contrast to stress paradigms, ethanol administration alters glutamate and GABA transmission in a region-specific manner. Acute ethanol administration dose-dependently increased (0.5 g/kg, i.p.) or decreased (1.0–2.0 g/kg, i.p.) extracellular glutamate in the striatum and hippocampus (Moghaddam and Bolinao, 1994). While direct evidence of ethanol’s effect on parvocellular neurons in the PVN remains unknown, previous work has demonstrated that chronic ethanol consumption in rats results in a blunted HPA axis activation response to microinjection of picrotoxin, a GABA_A_ receptor antagonist (Li et al., 2011).

Adrenal steroid hormones have neuronal membrane receptors that regulate neurotransmission and alter neural activity. For example, eliminating all adrenal hormones via adrenalectomy increased the number of GABAergic contacts onto CRH neurons in the PVN (Miklós and Kovács, 2002). Adrenalectomy also resulted in attenuation of extracellular glutamate in the hippocampus and prefrontal cortex (Moghaddam et al., 1994), two regions with important regulatory roles for HPA axis activity. Ethanol’s stimulating effect on HPA function is apparently diminished with repeated exposure. For examples, blunted plasma cortisol levels, both basal and pharmacologically stimulated, have been reported in human alcoholics (Adinoff et al., 2005). In cynomolgus macaques, chronic ethanol self-administration was also associated with reduced basal cortisol (Helms et al., 2012a,b) and, interestingly, increased in these same monkeys during prolonged abstinence (Cuzon Carlson et al., 2011). ACTH was also dampened by chronic ethanol self-administration in monkeys (Helms et al., 2014). In contrast, the neuroactive and mineralocorticoid precursor deoxycorticosterone was increased after chronic ethanol self-administration in male rhesus macaques (Helms et al., 2014). Overall the data indicate that adrenal steroid hormones are altered during long-term ethanol self-administration in primates, but the contribution and/or impact on neurotransmission involving the PVN and response to stress is unknown. However, because the balance of excitatory and inhibitory signals in the PVN is critical for maintaining appropriate control over homeostatic processes during stress, this balance may be key to unraveling the pathological interaction of ethanol and stress.

We utilized a non-human primate model of ethanol self-administration to investigate presynaptic signaling between the primary neurotransmitters GABA and glutamate and the primary secretagogues of the HPA axis, CRH and AVP. These data are the first to describe the unique relationship between chronic ethanol self-administration and the relative GABA and glutamate density in presynaptic terminals within the primate PVN. Additionally, the results from these studies revealed two populations of axon terminals with distinct relationships to stress, ethanol and fluid homeostasis.

## Materials and Methods

### Animals

Young adult female rhesus macaques (*Macaca mulatta*) between the ages of 5.5–6.0 years at the onset of the study were subjects in the current experiment. Animals were assigned to one of two groups, ethanol (*n* = 5) or control (*n* = 3). All animals were housed in quadrant cages (0.8 × 0.8 × 0.9 m) with constant temperature (20–22°C), humidity (65%) and a 11-h light cycle (lights on at 08:00 AM) with visual, auditory and olfactory contact with other conspecifics. In addition to 2 h/weekday when the barrier between monkeys housed side-by-side was removed and the monkeys shared the expanded housing cage. Body weights were measured weekly. All procedures were conducted in accordance with the Guide for the Care and Use of Laboratory Animals and approved by the Oregon National Primate Research Center—Institutional Animal Care and Use Committee (IACUC).

### Blood Collection and Mild Stress-Induced Activation of the HPA Axis

After acclimating to the laboratory and staff, training for awake venipuncture was performed twice daily and advanced for each animal individually as they readily performed each step with minimal observable distress. Training was conducted by presenting fresh fruit when the animal sat at the front of the cage and presented a leg through an opening in the housing cage (10 × 10 cm). Once the animal reliably presented a leg, a dental pick was used to simulate a needle stick. Finally, a 3-ml blood sample was collected through a 22-gauge needle into a serum-separator Vacutainer tube (BD, Becton Dickinson) from the femoral vein. After blood could be obtained from each subject the animals were trained to sit in a primate chair. Briefly, a pole was attached to the animal’s collar, which was used to guide the animal to the chair, where blood could also be obtained for plasma hormone assay. After training, removing the monkey from the cage and into the chair is a mild stressor as indicated by slightly elevated cortisol (Ruys et al., 2004). Chair training was completed in 11 training sessions. These two locations (home-cage or chair) served as distinct conditions (low and mild stress, respectively). Blood collection for plasma hormone assay from the home-cage occurred three times per week for 8 months prior to the collection of samples assayed for the current project. Hormonal response to these two conditions were measured twice: after the animals had been trained to obtain all food and fluids from the operant panel and again after approximately 12 months of ethanol self-administration, 30 days before tissue collection.

### Plasma Assays

All blood samples were collected within the first 3 h of the onset of the light cycle, and stored on ice for approximately 15 min until centrifuged for 15 min at 4°C (Beckman Colter, Model Allegra 21R). Plasma was aliquoted and stored at −80°C until assayed for cortisol, ACTH, aldosterone and sulfated DHEA (DHEA-s). Assays were conducted by the Oregon National Primate Research Center Endocrine Technology Services Laboratory. A Roche Cobas e411 automatic clinical platform was used to assay ACTH (1–2000 pg/ml sensitivity, 0.8% inter-assay variation), cortisol (0.036–63.4 μg/dl sensitivity, 1.1% inter-assay variation) and DHEA-s (sensitivity, 0.001–10 μg/ml; inter-assay variation, 4.4%). Aldosterone was measured using commercially available enzyme-linked immunosorbent assays with sensitivity of 0–1600 pg/ml and inter-assay variation of 7.8%. Blood (20 μl) was collected from the saphenous vein every fifth day, 7 h into the daily session, for analysis of blood ethanol concentration (BEC) using gas head-space chromatography (Hewlett-Packard 5890 Series II, Avondale, PA, USA; equipped with a headspace auto-sampler, flame ionization detector, and a Hewlett Packard 3392A integrator). Blood was added to 500 μl of sterile water and 20 μl of isopropyl ethanol, an internal standard, and frozen in a sealed glass vial until assayed. Duplicate standards of known concentration ranging from 25–400 mg/dl were used to generate a standard curve. Standards and samples were assayed on the same day.

### Ethanol Access

Details of the operant panel have been previously described (Vivian et al., 2001; Grant et al., 2008). Briefly, an operant panel was attached to one wall of the animals’ home cage and dispensed all food and fluids. Each panel had two sets of three lights (white, red and green) that signaled an active session, food and fluid availability, respectively. Below each light set was a fluid spout that was activated by pulling a recessed dowel located near the center of the panel. Nalgene tubing connected each fluid spout to a 1-L fluid reservoir that sat on a digital scale (Ohaus Navigator Balances N1B110, Ohaus Corporation, Pine Brook, NJ). An infrared finger-poke while pulling the dowel resulted in the delivery of a 1-g banana pellet (BioServe, Flemington, NJ, USA). Dowel pulls, finger pokes and fluid consumption were recorded every 500-ms via a computerized system using custom hardware and programing (National Instruments interface and Labview Software, Austin, TX, USA). Ethanol access began with schedule-induced polydipsia as described by Grant et al., 2008. During induction a 1-g banana food pellet was delivered every 300-s (FT-300) until a predetermined volume of fluid was consumed, after which water was freely available and after 2 h any remaining pellets were available on a FR-1 schedule. The first phase of the induction procedure, which lasted 54 consecutive days, requires each animal to consume a water equivalent of 1.5 g/kg of 4% ethanol. Next animals were required to consume 4% (w/v) ethanol in 30-day increments to meet a daily dose equal to 0.5, 1.0 and 1.5 g/kg. Following induction, animals were given concurrent access to 4% ethanol and water for 22-h per day. During this time pellets were available in three equal meals separated by 2 h on a FR-1 schedule. During the 2-h break between daily sessions data was downloaded, husbandry tasks were performed, animals were pair-housed and fresh fruit and vegetables were provided. A total of 389 consecutive open access sessions were completed. Fourteen days were removed when calculating the average daily ethanol consumption due to sedation or shortened sessions, however ethanol consumed on these days is included in the lifetime consumption.

### Necropsy and Tissue Collection

**Figure 1:**
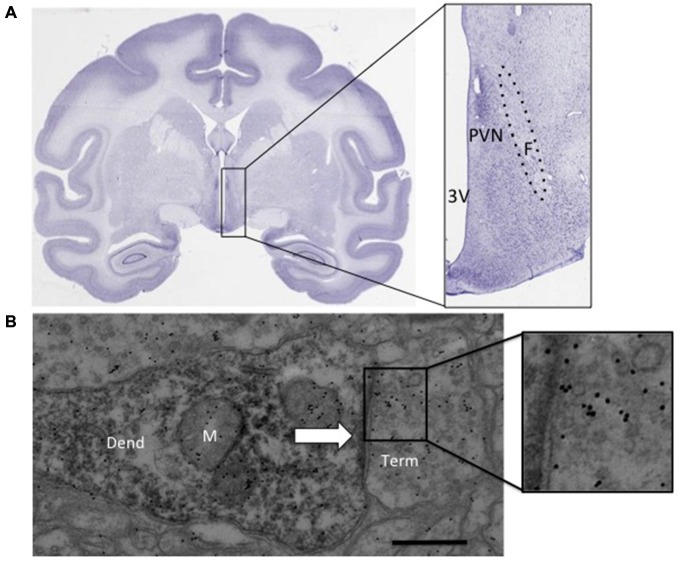
**(A)** Nissl stained coronal section from an adult rhesus macaque brain (BrainMaps, 2014). The inset shows the approximate region dissected. **(B)** Immunoreactive dendrite with synaptic contact (arrow), inset shows immunogold labeling above synaptic vesicles. paraventricular nucleus (PVN): PVN of the hypothalamus, F, fornix; 3 V, third ventricle; Dend, dendrite; M, mitochondria; Term, synaptic terminal. Scale bar 500 nm.

Each animal was sedated at what would have been the start of that day’s drinking session. The final session was identical to all others and each animal consumed water and ethanol in a normal pattern. Each animal was sedated with 10 mg/kg ketamine, intubated, and maintained with isoflurane and pentobarbital. A craniotomy was performed and immediately followed by perfusion with oxygenated artificial cerebral spinal fluid stored on wet ice. The perfusion was complete within 4 min and the brain was immediately removed. The brain was dissected into 4-mm coronal sections using a brain block (TedPella, Inc, Redding, CA, USA), as described by Daunais et al., 2010. The hypothalamic PVN was removed and both hemispheres of a single 4 mm block were placed into fixative. The approximate area dissected is shown as the inset in Figure 1A (BrainMaps, 2014). The left hemisphere was fixed for electron microscopy (EM) by fixing the tissue in 2.5% gluteraldehyde, 0.1% picric acid and 0.5% paraformaldehyde solution (in 0.1 M phosphate buffer) for 24 h, then 4% paraformaldehyde for 24 h and stored in 0.1 M phosphate buffer until sectioned. The right hemisphere was fixed in four percentage paraformaldehyde for 48 h then 30% sucrose until sectioned and used for light microscopy.

### Electron Microscopy Processing and Analysis

A vibratome was used to collect 60 μm sections (Leica Microsystems, Buffalo Grove, IL, USA). Five sections containing the PVN were chosen in which the fornix was fully extended (Figure 1A). The selected tissue sections were processed for EM using pre-embed diaminobenzidine (DAB) immunohistochemical labeling for localization of AVP (Millipore, 1:4000, rabbit) or CRH (Santa Cruz, 1:500, rabbit) using a modified microwave procedure (Walker et al., 2012). Tissue was incubated in the microwave (Pelco BioWave, TedPella, Inc., Redding, CA, USA) for 5 min, 550 W, at 35°C with the vacuum off (all the remaining steps occurred at this temperature) with the vacuum cycling down to 20 Hg, then back to atmosphere repeatedly in 10 mM sodium citrate, pH 6.0 (antigen retrieval), rinsed in 0.1 M phosphate buffer saline (PBS) for 1 min at 150 W with the vacuum off, exposed to 3% hydrogen peroxide at 150 W for 1 min with the vacuum off, rinsed in PBS at 150 W for 2 × 1 min with the vacuum off, exposed to 0.5% Triton X-100 for 5 min, 550 W with the vacuum cycling, then exposed to one of the primary antibodies for 48 h at 4–5°C. The tissue was then rinsed in PBS 1 min × 2 each at 150 W with the vacuum off, then exposed to the secondary antibody (bioatinylated goat anti-rabbit, 1:100; Vector Laboratories, Burlingame, CA, USA) for 16 min at 200 W for two cycles of the following: 4 min on, 3 min off, 4 min on, 5 min off, all on a continuous vacuum (20 Hg). The tissue was then rinsed in PBS for 1 min, followed by a rinse in working imidazole buffer (0.01M imidazole, 0.016M Na Acetate aqueous) at 150 W with the vacuum off and then exposed to avidin-biotin complex (ABC; Vector Elite Kit, 1% solution A and B in working imidazole buffer) for 16 min at 200 W under constant vacuum using the following cycle: 4 min on, 3 min off, 4 min on, 5 min off. The tissue was then rinsed in working imidazole buffer twice at 1 min each, at 150 W with the vacuum off and then exposed to DAB (0.5 mg/ml + 1.5% hydrogen peroxide) for 10 min under constant vacuum at 200 W. The tissue was embedded in Epon-Spurs at 60°C for approximately 16 h. The region of interest (2 mm dorsal-ventral between the ventricular edge and the fornix) was dissected and mounted onto a resin block for thin sectioning. Sections were cut (60 nm) on an ultra-microtome (EM UC7; Leica Microsystems, Buffalo Grove, IL, USA) using a diamond knife (Diatome, Hartford, CT, USA) and collected onto 100 mesh formvar covered grids (EM Sciences, Hatfield, PA, USA). Post-embed immunogold EM was performed using a glutamate (non-affinity-purified, rabbit polyclonal; G-6642 purified glutamate conjugated to KLH as the immunogen, Sigma-Aldrich, St. Louis, MO, USA) and GABA (A2052 antibody is isolated from antiserum by immune-specific methods of purification, Sigma-Aldrich, St. Louis, MO, USA) on adjacent thin sections that had been previously labeled with DAB for CRH or AVP. The primary glutamate antibody, as described previously (Phend et al., 1992), diluted 1:250 in Tris-buffered saline with Tween 20 (TBST) 7.6 and aspartate (1 mM) was added to the glutamate antibody mixture 24 h before incubation with the thin-sectioned tissue to prevent any cross-reactivity with aspartate. The GABA antibody was diluted in TBST pH 8.2 immediately prior to use. The secondary antibody for both GABA and glutamate was goat anti-rabbit IgG conjugated with 12 nm gold particles (diluted 1:50 in TBST, pH 8.2; Jackson ImmunoResearch, West Grove, PA, USA). Meshul et al. (1994) previously demonstrated the specificity of the glutamate and GABA antibodies using a competition assay in which incubation of the antibody with 3 mM of glutamate or GABA, respectively, resulted in no immunogold labeling.

Electron micrographs (50–60/animal) were taken on a JEOL 1400 transmission electron microscope of presynaptic terminals contacting DAB-labeled postsynaptic structures at a final magnification of 8000× using a digital camera (Figure 1B, Advanced Microscopy Techniques, Woburn, MA, USA). For quantification of immunogold labeling, the relative density of gold particles per square micrometer of nerve terminal area was determined using Image-Pro 6.3 software (Media Cybernetics, Inc., Rockville, MD, USA). The mean density for each animal and group was calculated. Background labeling was determined within glial cell processes and was found to be approximately 10 immunogold-labeled particles/μm^2^ (Meshul et al., 1994). This was subtracted from the density of presynaptic immunogold-labeled glutamate within the nerve terminals.

### Light Microscopy Processing and Analysis

Primary antibodies for both CRH and AVP were produced in rabbit. In order to perform double-label immunohistochemistry the CRH antibody was conjugated to biotin using the following protocol: 500 μl of CRH anti-rabbit primary antibody (Santa Cruz Biotechnology, Inc., sc-10718) was concentrated using a VivaSpin500 centrifuge tube (Satorius, Bohemia, NY, USA) for 10 min at 15000 g. The concentration of antibody was measured using NanoDrop spectrophotometer and diluted in PB to ~1 mg/ml. A Biotin-XX kit (Molecular Probes, Grand Island, NY, USA) was used to covalently link the concentrated CRH antibody to biotin. Sections were collected using a freezing microtome. Six 30 μm serial free-floating sections (180 μm distance apart) from each animal were incubated in the microwave (Pelco BioWave, TedPella, Inc, Redding, CA, USA) for 5 min, 550 W, at 35°C with the vacuum off (all the remaining steps occurred at this temperature) in 10 mM sodium citrate, pH 6.0 (antigen retrieval), rinsed in 0.1 M phosphate buffer (PB) for 2 × 1 min at 150 W with the vacuum off, exposed to 3% hydrogen peroxide at 150 W for 1 min with the vacuum on, rinsed in PB at 150 W for 2 × 1 min with the vacuum off, exposed to 0.5% Triton X-100 for 5 min, 550 W with the vacuum on, washed in PB for 2 × 1 min at 200 W with the vacuum off, then exposed to the AVP primary antibody (Millipore, ab1565; 1:4000) for 48 h at 4–5°C. All remaining steps were done on the bench-top. Tissue was rinsed in PB (4 × 5 min, 3 × 2 min, 2 × 20 s rinses). Secondary (Alexa Fluor 488 donkey anti-rabbit, 1:1000) was applied at room temperature for 1 h on a rotating plate while protected from light. The tissue was rinsed in PB as described previously then incubated in biotinylated CRH primary antibody (1:500) for 72 h in the refrigerator on a rotating plate while protected from light. The tissue was rinsed in PB prior to use of a Tyramide Signal Amplification kit (Molecular Probes, T20936) using a streptavidin-linked Alexa Fluor 647 sary antibody. All sections were counterstained using Hoeschst (Life Technologies, Grand Island, NY, USA; 1:15,000 in PB) for 1 min before the final set of rinses. Tissue sections were mounted onto slides and cover-slipped with 200 μl ProLong Gold anti-fade mounting reagent (Life Technologies, Grand Island, NY, USA). Additional tissue sections were processed in the absence of primary antibody to confirm secondary antibody specificity.

Images were acquired using a Marianas imaging workstation (Intelligent Imaging Innovations, Denver, CO, USA), using Slidebook 5.5. Excitation light was provided by a DG-4 fluorescence illumination system (Visitron Systems GmbH) and filtered through a Sedat Quad set (Chroma Technology) and detected by a CoolSNAP HQ CCD camera (Photometrics). A 10 × NA 0.45 Plan-Apochromat objective was used to acquire and construct a large montage of 3 × 5 fields of view. All image acquisition parameters for each channel, including the exposure times and the histogram domain selected for display and tiff export were kept constant throughout the experiment: 500 ms in fluorescein iso-thiocyanate (FITC) channel captured AVP signal, 20 ms exposure in 4′,6-diamidino-2-phenylindole (DAPI) captured the nucleic marker, 500 ms in the Cy3 channel captured CRH signal and 900 ms in the Cy5 channel to capture nonspecific background autoflorescence that was subtracted from the AVP and CRH images during analysis. Images were analyzed in ImageJ (Rasband and Image, 1997–2014). For each section, the region of interest was outlined as the area of densely packed fluorescently labeled cells, and applied to each channel. The mean intensity (arbitrary units optical density, O.D.) for each channel was measured in six serial sections and used to calculate a mean intensity for each animal which was then used to calculate a group mean.

### Statistics

Changes in circulating stress hormones were analyzed with a general linear model with repeated measures using a between-subject factor of group (controls and drinkers) and within-subject factors of experimental phase (baseline and post self-administration) and condition (low or mild stress). Group comparisons were made between density parameters and immunofluorescence using independent Student’s *t*-test. Pearson’s correlation was used to assess relationships between GABA and glutamate immunogold density, OD, hormone concentrations and fluid consumption. Fluid consumption is reported in g/kg for ethanol and ml/kg for water in order to normalize relative to body weight. In order to capture a period of ethanol intake that may be more directly related to the ultrastructural changes, the last month of self-administration was analyzed independently. All values are reported as mean ± standard deviation (SD). All tests were two-tailed and α < 0.05 was considered significant. Analyses were conducted using Statistical Package for Social Sciences (SPSS) version 21 (Armonk, NY, USA).

## Results

### Ethanol Self-Administration and Blood Ethanol Concentration

Table 1 lists the average daily intake (g/kg/day ± SD) for the 375 consecutive sessions and final 30 days of ethanol self-administration. Average daily intake did not differ during the last month of access compared to the total open access average intake (*p* > 0.05). Longitudinal BECs (BEC, *n* = 61/animal) at 7 h into the 22 h session was found to highly correlate with the average intake at the time of the sample; *r* = 0.88, *p* = 0.018.

**Table 1:** **Average daily ethanol consumption over the duration of open-access conditions (375 days) and the last 30 days of access**.

	Ethanol (g/kg/day)	
Animal ID	All days	Last 30 days
10072	1.1 (0.5)	1.2 (0.4)
10077	1.3 (0.6)	0.9 (0.3)
10074	1.7 (0.8)	1.6 (0.8)
10075	2.7 (1.1)	2.3 (0.6)
10073	4.0 (1.2)	4.6 (0.7)

*All values are mean g/kg/day (± SD)*.

### Effect of Ethanol Self-Administration on Circulating Hormones in Low and Mildly Stressed Conditions

**Figure 2:**
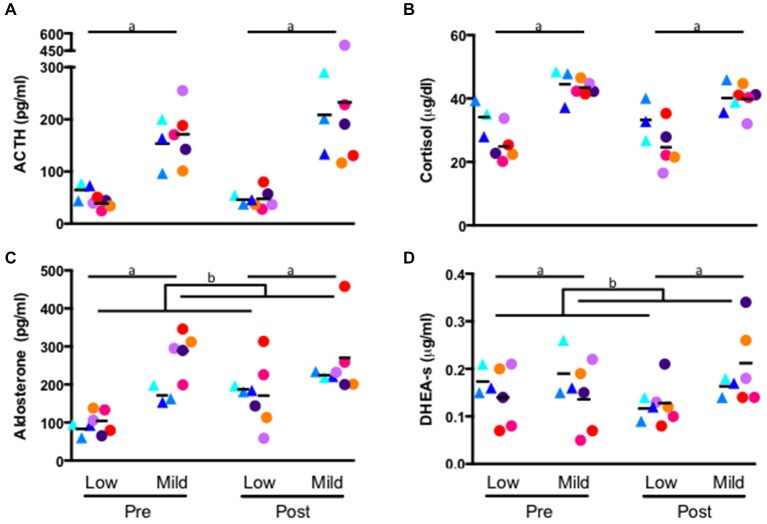
**Hormone concentration of ACTH (A) cortisol (B) aldosterone (C) and DHEA-s (D) measured from plasma collected in the home-cage (low stress) and from a primate chair (mild stress)**. Samples were assayed prior to self-administration (pre), and following self-administration (post). (a) All hormone concentrations were significantly higher in response to the mild stress condition (*p* > 0.05) for both groups at baseline and following self-administration. (b) Experimental phase by condition interaction (*p* > 0.01), see text. Triangles: controls, circles: drinkers. Each animal is indicated with a unique color.

A repeated measures general linear model revealed a significant effect of stress condition on each hormone: ACTH (low stress: 47.80 ± 17.34 pg/ml, mild stress: 194.17 ± 88.64 pg/ml; F_1,*6*_ = 18.16, *p* = 0.005, Figure 2A), cortisol (low stress: 28.12 ± 7.40 μg/dl, mild stress: 41.91 ± 4.15 μg/dl; F_1,*6*_ = 56.17, *p* < 0.001, Figure 2B), aldosterone (low stress: 137 ± 52 pg/ml, mild stress: 249 ± 80 pg/ml; F_1,*6*_ = 31.88, *p* = 0.001, Figure 2C) and DHEA-s (low stress: 0.139 ± 0.048 μg/ml, mild stress: 0.174 ± 0.070 μg/ml; F_1,*6*_ = 19.24, *p* = 0.005, Figure 2D). Each hormone was significantly elevated during the mildly-stressed (chaired) condition. In addition to the main effect of stress condition, several interactions emerged. A significant interaction of experimental phase and condition where aldosterone during self-administration was greater than baseline for all animals, but the magnitude of increase was greater in response to the low stress compared to the mild-stress condition (low stress: baseline: 96 ± 29 pg/ml, post self-administration: 177 ± 76 pg/ml; Mild-stress: baseline: 245 ± 74 pg/ml, post self-administration: 253 ± 85 pg/ml F_1,*6*_ = 12.09, *p* = 0.01, Figure 2C), although the interaction between stress condition and group did not reach significance (*p* = 0.07). A significant interaction between stress condition and experimental phase indicated that DHEA-s was lower after self-administration in the low stress condition, but increased following self-administration in response to the mild-stressor (baseline: low stress: 0.154 ± 0.057 μg/ml, mild stress: 0.156 ± 0.071 μg/ml; post self-administration: low stress: 0.125 ± 0.040 μg/ml, mild stress: 0.194 ± 0.071 μg/ml; F_1, *6*_ = 16.04, *p* = 0.007, Figure 2D), although there was no significant interaction with group (F_1,*6*_ = 3.84, *p* = 0.10). Controls and drinkers did not differ significantly in cortisol, although there was a trend towards an interaction of group and condition where monkeys in the ethanol group had lower concentration of cortisol during the low stress condition compared to controls (controls: 33.69 ± 6.19 μg/dl, drinkers: 24.78 ± 6.24 μg/dl; *p* = 0.06) and in response to the mild-stressor (controls: 42.34 μg/dl ± 5.80 μg/dl, drinkers: 41.66 μg/dl ± 3.39 μg/d; F_1,*6*_ = 5.83, *p* = 0.05, Figure 2B).

Percent change for each hormone, calculated as (mild-stress—low stress)/low stress*100, for each experimental phase was compared using a paired *t*-test. A significant percent change in DHEA-s was elicited by the mild-stress, relative to the low stress condition, in drinkers following ethanol self-administration (−8 ± 15% at baseline and 65 ± 30% following ethanol self-administration; *T*_4_ = −5.2, *p* = 0.006, Figure 2D), but not controls (7 ± 13% at baseline and 41 ± 11% following the self-administration phase; *T*_2_ = −2.6, *p* = 0.12). A trend-level difference in aldosterone response to the mild stress relative to the low stress condition was found for controls (114 ± 53% at baseline and 20 ± 9% following self-administration; *T*_2_ = 3.40, *p* = 0.077, Figure 2C) but was not present in drinkers (206 ± 129% at baseline, 95 ± 115%; *T*_4_ = 1.38, *p* = 0.24). No significant differences were found in percentage change of cortisol or ACTH in either group.

### Effect of Ethanol on Relative GABA Density

**Figure 3:**
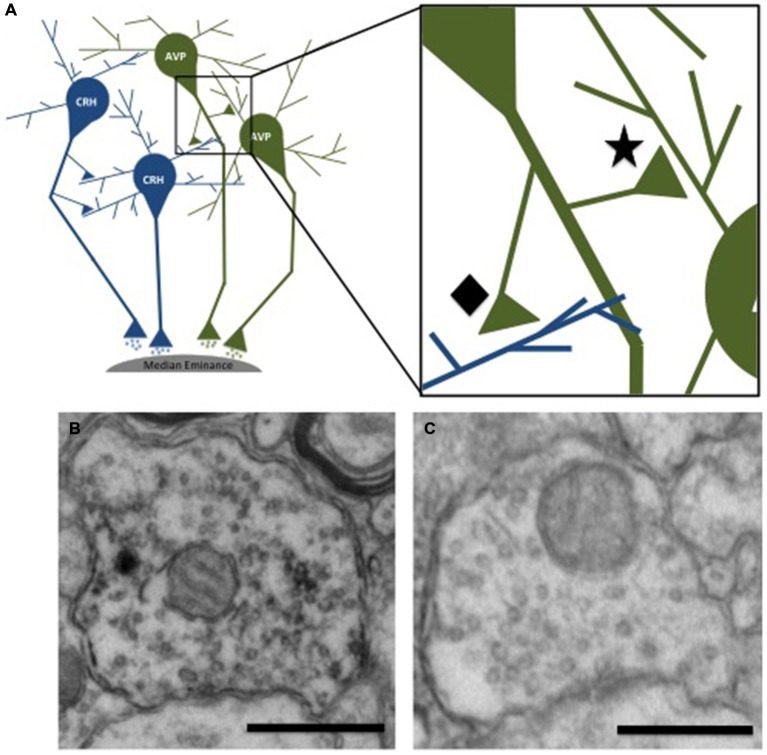
**Schematic illustration of neurons in the PVN projecting to the median eminence**. Recurrent axon collaterals can be seen contacting neighboring parvocellular neurons. **(A)** The inset shows recurrent terminals in which both the nerve terminal and postsynaptic dendrite are immunoreactive for the same peptide (★) and non-recurrent synaptic contacts where the nerve terminal and postsynaptic dendrite are not immunoreactive for the same neuropeptide (⧫) Electron micrographs illustrating the two types of presynaptic terminals. The presynaptic terminal shown in **(B)** is immunoreactive for vasopressin, as indicated by the electron dense DAB staining of the vesicles. The terminal shown in **(C)** is not immunoreactive for vasopressin. Scale bar 500 nm.

**Figure 4:**
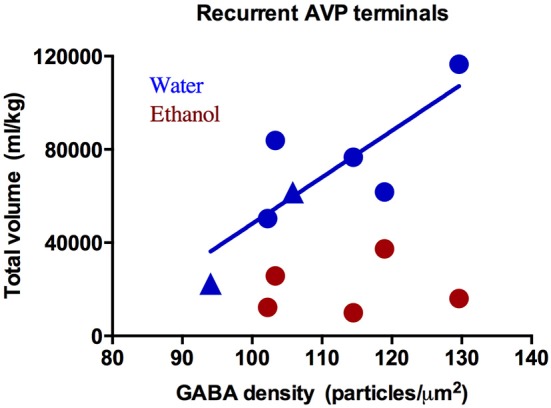
**GABA immunogold density (particles/μm^2^) in recurrent arginine-vasopressin (AVP) axon collaterals correlates with water intake (blue: *R*^2^ = 0****.82, *p* = 0.03) but not ethanol (red: *R*^2^ = 0.10, *p* = 0.88). Circles: drinkers, triangles: controls**. Only two of the three controls were included in analysis of immunogold density and AVP due to a technical error in tissue processing.

The presynaptic density of GABA and glutamate was measured in presynaptic terminals contacting immunohistochemically-identified CRH or AVP dendrites. This analysis revealed two populations of axon terminals: those labeled with a neuropeptide (CRH or AVP; Figure 3B) and those that did not (Figure 3C). Few regions, particularly those immunoreactive for CRH and AVP, have direct projections to neurons in the PVN, but instead information is relayed primarily via GABAergic and glutamatergic peri-PVN and hypothalamic regions to the PVN (Herman et al., 2005). Recurrent axon collaterals (locally synapsing terminals originating from parvocellular neurons in the PVN illustrated in Figure 3A) have been previously reported and are likely these CRH- and AVP-labeled terminals (van den Pol, 1982; Ray and Choudhury, 1990). Table 2 lists the mean GABA and glutamate immunogold density. Presynaptic GABA density did not differ by group, nor did it correlate with average daily ethanol intake (g/kg/day) over 12 months (CRH: *r* = 0.45, *p* = 0.26; AVP: *r* = 0.47, *p* = 0.29) or the last 30 days (CRH: *r* = 0.16, *p* = 0.80; AVP: *r* = 0.09, *p* = 0.88). GABA density in recurrent AVP axon collaterals was found to correlate with the volume (ml/kg) of water (*r* = 0.82, *p* = 0.025), but not ethanol (*r* = 0.10, *p* = 0.88) consumed during self-administration, Figure 4. Analysis of both GABA and glutamate density related to AVP was limited to two ethanol-naïve controls due to inadequate resin infiltration during tissue processing.

**Table 2:** **Mean (± SD) GABA and glutamate density in control (*n* = 3) and ethanol-drinking monkeys (*n* = 5)**.

Neurotransmitter	Neuropeptide	Contact type	Mean density (particles/μm^2^) controls*	Mean density (particles/μm^2^) drinkers	*p*-value
GABA	AVP	Recurrent	99.9 (8.3)	113.7 (11.4)	0.19
		Non-AVP	130.3 (19.8)	102.5 (32.0)	0.32
	CRH	Recurrent	118.7 (27.8)	164.3 (41.1)	0.15
		Non-CRH	107.0 (16.2)	143.8 (40.2)	0.31
Glutamate	AVP	Recurrent	48.8 (2.0)	71.6 (23.5)	0.10
		Non-AVP	77.4 (12.0)	87.9 (24.4)	0.60
	CRH	Recurrent	86.2 (20.5)	87.1 (18.2)	0.97
		Non-CRH	79.5 (22.4)	80.1 (16.8)	0.95

Recurrent contacts refer to terminals that originate in the PVN as indicated by immunoreactive staining for the same neuropeptide as the post-synaptic dendrite. *Controls used in AVP analysis, *n* = 2; controls used in CRH analysis, *n* = 3.

### Effect of Ethanol on Relative Glutamate Density

**Figure 5:**
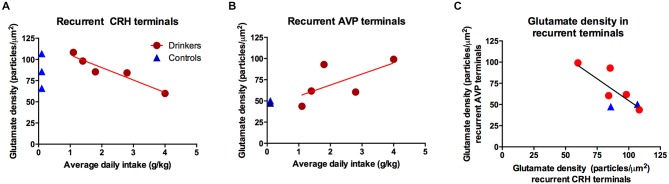
**The glutamate immunogold density in (A) recurrent corticotropin-releasing hormone (CRH) terminals was highly correlated with the average daily ethanol intake over open-access conditions (*R*^2^ = −0.91, *p* = 0.01); however there was no group difference *p* = 0.97, as illustrated in the inset displaying the group means**. **(B)** Recurrent AVP terminals did not correlate with average daily ethanol intake (*R*^2^ = 0.65, *p* = 0.23), a weak trend towards an increase in animals consuming ethanol as seen in the inset (*p* = 0.10). **(C)** Glutamate density in CRH and AVP recurrent terminals were correlated (*R*^2^ = −0.78, *p* = 0.04). Blue triangles: controls; red circles: drinkers.

No group differences were found in glutamate density. Although the range of glutamate density in ethanol animals was similar to controls, the glutamate density in co-labeled CRH terminals was linearly related to average daily ethanol intake (*r* = −0.91, *p* = 0.012), Figure 5A. As expected, the last 30 days revealed a significant correlation between glutamate density in recurrent CRH axon collaterals and volume (ml/kg) of ethanol consumed (*r* = −0.92, *p* = 0.026), but not water (*r* = 0.14, *p* = 0.74), given that the average daily intake did not differ between the entire open-access condition and the last 30 days.

Conversely, glutamate density in recurrent AVP axon collaterals was not correlated with 12-month average daily ethanol intake (g/kg; *r* = 0.65, *p* = 0.23, Figure 5B). When considering only the last 30 days, no significant correlations were found between the glutamate density in recurrent AVP terminals and total ethanol consumed (ml/kg; *r* = 0.66, *p* = 0.23) or total water consumed (ml/kg; *r* = 0.35, *p* = 0.45). The glutamate density in non-recurrent AVP or CRH terminals did not correlate with total volume of water or ethanol consumed over the 12 months of self-administration. The glutamate density in recurrent AVP and CRH axon collaterals was significantly correlated (*r* = −0.78, *p* = 0.04; Figure 5C), suggesting a coordinated balance of excitatory input onto these principal cell populations. Among recurrent AVP axon collaterals there was a trend for glutamate and GABA density to be correlated (*r* = 0.72, *p* = 0.07), however this was not the case for CRH (*r* = 0.09, *p* = 0.82).

### Effect of Ethanol Self-Administration on CRH and AVP: Relationship with GABA and Glutamate Density and Circulating Stress Hormones

**Figure 6:**
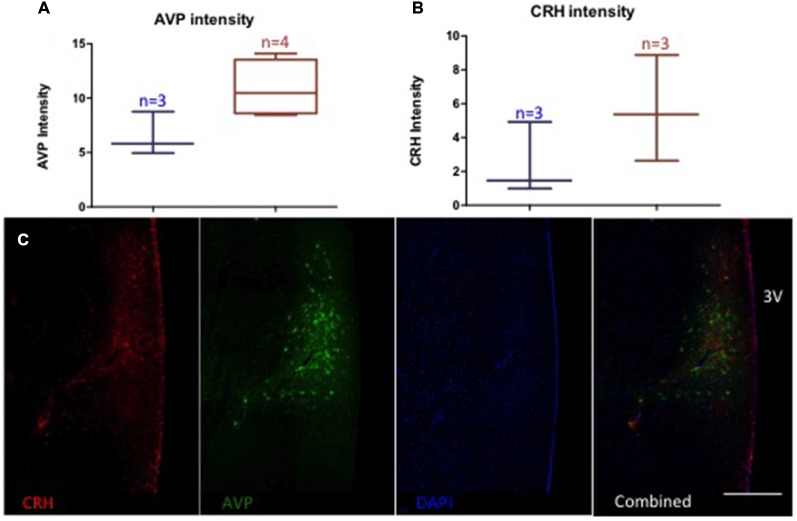
**Relative intensity (optical density, arbitrary units) of AVP (A) and CRH (B)**. A trend towards increased AVP optical density in animals consuming ethanol (red) compared to ethanol naïve (blue) (*p* = 0.06), but no difference in CRH (*p* = 0.22). **(C)** Representative image of immunolabeling. 3 V: third ventricle. Scale *bar* = 500 μm.

Monkeys chronically consuming ethanol showed a trend towards elevated AVP-immunoreactivity (controls (*n* = 3): 6.5 ± 2.0 O.D., drinkers (*n* = 4): 10.9 ± 2.6 O.D., *p* = 0.06) and no difference in CRH (controls (*n* = 3): 2.5 ± 2.2 O.D., drinkers (*n* = 3): 5.6 ± 3.1 O.D., *p* = 0.22), Figure 6. Glutamate density in recurrent AVP terminals weakly correlated with the OD of AVP (*r* = 0.71, *p* = 0.06). AVP immunoreactivity was correlated with the percent change in aldosterone from baseline to chronic ethanol self-administration conditions under the mildly-stressed condition (*r* = −0.77, *p* = 0.04) but not under the low-stress condition (*r* = −0.20, *p* = 0.67). The percent change in ACTH in the low-stress condition reached trend-level significance with AVP immunoreactivity (*r* = 0.73, *p* = 0.06). CRH immunoreactivity and the percent change of DHEA-s over time significantly correlated with both mildly-stressed (*r* = 0.82, *p* = 0.047) and low-stress (*r* = 0.92, *p* = 0.008) conditions and weakly correlated with percent change of cortisol in low-stressed condition (*r* = 0.76, *p* = 0.08). Thus, AVP immunoreactivity was relatively more associated with aldosterone and ACTH response to stress and ethanol consumption and CRH was relatively more responsive to DHEA-s and cortisol response to ethanol consumption.

### Circulating Stress Hormones and GABA and Glutamate Density

Selective correlations between presynaptic GABA or glutamate density and the change in hormone response following self-administration emerged for both the low- and mildly-stressed conditions. GABA density in recurrent CRH terminals significantly correlated with the percent change in ACTH in the low stress (*r* = 0.73, *p* = 0.039), but not the mildly-stressed condition. GABA density in recurrent CRH terminals also significantly correlated with percent change in DHEA-s following self-administration only in the mildly-stressed condition (*r* = 0.90, *p* = 0.002), while only reached trend level in the low stress condition (*r* = 0.68, *p* = 0.06). No correlations were found with GABA density in non-recurrent CRH terminals. Glutamate density in recurrent CRH terminals correlated with the percent change in ACTH following self-administration in the mildly-stressed (*r* = −0.77, *p* = 0.024), but not the un-stressed condition. No other hormonal changes reached significance with GABA or glutamate density in terminals contacting CRH-immunoreactive dendrites. Glutamate density in non-recurrent terminals contacting AVP dendrites were significantly correlated with the percent change in cortisol for both low (*r* = −0.85, *p* = 0.016) and mild (*r* = −0.78, *p* = 0.04) stress conditions. Additionally, the percent change in aldosterone significantly correlated with glutamate in non-recurrent AVP terminals in the low-stress condition (*r* = −0.81, *p* = 0.029). Under mild stress the percent change in aldosterone following self-administration reached trend-level significance with the glutamate density in both recurrent (*r* = −0.70, *p* = 0.08) and non-recurrent AVP (*r* = −0.67, *p* = 0.10) terminals.

## Discussion

The most important finding presented here is the unique positive relationship of chronic ethanol intake and the relative glutamate density in recurrent axon terminals of the hypothalamic PVN. Recurrent axon collaterals have been found to originate from parvocellular neurons in both rats and monkeys (van den Pol, 1982; Rafols et al., 1987). The data presented here demonstrate that parvocellular neurons are uniquely related to ethanol intake, further implicating an interaction between the HPA axis at the level of the hypothalamus and ethanol consumption that may be a mechanism in which long-term ethanol consumption alters the activity of hypothalamic neurons, and ultimately alters HPA axis activity. Specifically, glutamate density in recurrent CRH terminals was highly correlated with average daily ethanol intake. Because the range of glutamate density measured after chronic ethanol drinking matches the range in ethanol-naïve controls, one interpretation is that PVN recurrent glutamate density is antecedent to ethanol exposure and may serve as a predictive factor in the development of heavy drinking. Nevertheless, the significance of glutamate density in terms of functional properties of PVN neurons requires further study. Glutamate density in the striatum measured by immunogold EM was negatively correlated with glutamate measured using *in vivo* microdialysis (See et al., 2002). Thus, EM can infer neurotransmitter release from terminals. However, it is unclear whether this is true in the PVN of the hypothalamus. In the present study glutamate density in recurrent CRH terminals was not correlated with pituitary-adrenal response under low or mildly stressed conditions, suggesting factors other than glutamate density in recurrent CRH terminals influence HPA response. Further, although the present study was an extensive investigation of pituitary-adrenal hormone response to stress including hormones from each of the three layers of the adrenal cortex, it was not exhaustive.

In addition to recurrent CRH axon terminals within the PVN, CRH-immunoreactive neurons originating in other brain regions, such as the bed nucleus of the stria terminalis (BNST) or amygdala, project to the PVN (Herman et al., 2003). These projections could contribute to the present results indirectly. For example, the BNST contains CRH-immunoreactive neurons however axon terminals from regions known to innervate the PVN, particularly the posterior division, were not found to be immunoreactive for CRH (Ju and Swanson, 1989). Few direct projections to the PVN have been observed (Ziegler and Herman, 2002). On a more global scale, single nucleotide polymorphisms in the CRH gene can influence HPA axis reactivity and sweetened ethanol consumption in non-human primates with early life stress (peer-rearing, Barr et al., 2009). Thus, it is possible that presynaptic glutamate density is a mechanism by which CRH signaling occurs within the PVN to predict future ethanol consumption, however, no studies to date have examined the effects of afferent CRH signaling on hypothalamic circuitry. CRH1R knockout mice have heightened ethanol intake after social defeat compared to wild-type mice, indicating CRH signaling modulates responses to stress related to ethanol self-administration (Sillaber et al., 2002). In contrast, using a conditional knockout in which CRH1Rs are preserved in the anterior pituitary and adrenal glands, thus maintaining the HPA axis response, social defeat did not affect ethanol consumption (Molander et al., 2012). These data in rodents suggest that CRH-dependent HPA axis signaling may mediate the effects of stress on ethanol self-administration, but this effect may be limited to particular experimental designs. The present data are the first to address mechanism(s) involving glutamate density in the primate PVN. Evidence for altered glutamate and GABA signaling after ethanol has been disproportionally focused on post-synaptic receptor changes following chronic ethanol consumption (see Mihic and Harris, 1997; Chandrasekar, 2013 for review) although more recently presynaptic effects have been gaining attention (see Siggins et al., 2005 for review).

Hypotheses have been made regarding vasopressin’s ability to regulate the HPA axis, particularly during adaptation to chronic stress (Aguilera and Rabadan-Diehl, 2000) and ethanol (Knott et al., 2000). The data here provides limited support for these hypotheses in that it appears elevated glutamate density in recurrent AVP axon collaterals may be a consequence of ethanol consumption because the current sample did not reach significance. Due to the small number of animals, particularly in the control group, these data should be interpreted with caution. These data do provide evidence of terminal- and neurotransmitter-specific interactions with vasopressin and ethanol that are independent of fluid balance. Specifically, GABA density in recurrent AVP axon terminals did not correlate with ethanol intake, but significantly correlated with water consumption. This finding was exclusive to AVP, as the GABA density in recurrent CRH terminals did not correlate with water or ethanol intake. The number of AVP-immunoreactive magnocellular neurons is reduced in rodents following 6 months of ethanol liquid diet (Silva et al., 2002). Interestingly, in these same animals AVP mRNA levels are unchanged, suggesting a compensatory upregulation within the remaining cells. Similar results were found in human alcoholics where the number of AVP-immunoreactive neurons and the volume of the PVN were negatively correlated with ethanol consumption (Harding et al., 1996). Based on the current data, one hypothesis is that increased glutamate density in a particular population of synapses (i.e., recurrent synapses) may be a possible mechanism for the increased activity of the post-synaptic neuron and ultimately maintenance of HPA axis activity. The positive trend-level correlations between glutamate density in recurrent AVP terminals with AVP immunofluorescence and aldosterone supports this proposed mechanism.

Blood samples routinely collected from the home cage show low levels of circulating stress hormones including ACTH, cortisol (Helms et al., 2014), DHEA-s and aldosterone (present study), consistent with the designation as a low stress condition. Together with the blood samples obtained from unanesthetized monkeys taken from the home cage and placed in a chair, these two conditions allowed for the effect of ethanol self-administration on resting and stimulated activity of the HPA axis to be analyzed. Elevations in ACTH, cortisol and DHEA-s are reflective of HPA axis activation (Smith and Vale, 2006; Maninger et al., 2010) while aldosterone has been related to fluid homeostasis (Booth et al., 2002) and cardiovascular function (Connell and Davies, 2005) consistent with increased levels in response to mild stress (Tucci et al., 1967; Raff and Chadwick, 1986). Ethanol self-administration in the present study did not disrupt the HPA response to a low or mild stress. Although we have previously reported decreased plasma cortisol in male cynomolgus monkeys (Cuzon Carlson et al., 2011; Helms et al., 2012a) the cortisol levels reported here in female rhesus monkeys did not change following ethanol consumption, similar to our previous findings in male rhesus monkeys (Helms et al., 2014). These data therefore confirm defense of this important circulating glucocorticoid during chronic ethanol self-administration in both male and female rhesus macaques. Overall the present data indicate that pituitary ACTH increased yet hormone response at each layer of the adrenal cortex was maintained over chronic ethanol self-administration, suggesting adrenal adaptation. The dissociation between ACTH and adrenal hormones was observed in male rhesus macaques for which correlations were absent after 12 months of ethanol access, again suggesting adrenal adaptation (Helms et al., 2014).

Independent of ethanol consumption, circulating DHEA-s decreased under the low stress condition but increased in response to the mild stress condition over time. These results are intriguing as both cortisol and DHEA-s modulate activity of parvocellular neurons. Cortisol acts as a negative feedback signal to both CRH and AVP parvocellular neurons (Erkut et al., 1998) while DHEA-s has been shown to modulate GABA and glutamate neurotransmission (Pérez-Neri et al., 2008, for review) and activate CRH and AVP parvocellular neurons in the PVN (Deuster et al., 2005; Naert et al., 2007). The present study was limited to an analysis of neurons, though an interaction of cell types could contribute to PVN signaling, as oligodendrocytes and astrocytes but not neurons synthesize DHEA, with its synthesis related to oxidative stress (Brown et al., 2000). The mechanisms within the PVN examined in the present study correlated only with increased DHEA-s response to mild stress, suggesting an effect of ethanol on specific steroidogenic pathways in the zona reticularis. GABA density in CRH terminals correlated with changes in DHEA-s response to stress after chronic ethanol self-administration, but the extent to which this represents a change in GABAergic transmission in specific cell populations of the PVN requires additional study. As adaptation at the level of the PVN would be expected to alter ACTH and subsequently all adrenal steroid hormones, the data suggest chronic ethanol self-administration has independent effects at the level of the adrenal.

Aldosterone is a mineralocorticoid induced principally by the renin-angiotensin system for maintaining fluid homeostasis (Connell and Davies, 2005). Dehydration or salt loading result in AVP produced by the magnocellular cells of the PVN, although some studies suggest that there is crosstalk between the magnocellular and parvocellular populations allowing the magnocellular neurons to contribute or modify the stress response (Engelmann et al., 2004). While the current study did not confirm the population of neurons responsible for the vasopressin content, it is unlikely the recurrent collaterals are of magnocellular origin as previous anatomical work has shown magnocellular neurons do not have axon collaterals (Rafols et al., 1987). However, the recurrent collaterals could synapse onto magnocellular dendrites, which have been shown to stay within the PVN-proper, thus contributing to the relationship with plasma aldosterone. Glutamate density within non-AVP terminals was related to circulating aldosterone concentrations in the low stress condition, suggesting that glutamate signaling in these specific cells may be a central mechanism regulating sensitivity to fluid imbalance. Indeed, a trend-level correlation with the immunogold density and vasopressin protein measured using immunofluorescence was found. Together a hypothesis that could be posited is that an upregulation of vasopressin, and perhaps vesicular glutamate, is a mechanism by which pituitary ACTH maintained response to stress during chronic ethanol self-administration, orthogonal to the contribution of CRH to the stress response.

Given that inhibition of GABA_A_ receptors in the PVN decreases ethanol self-administration in a rat model, implying an important role for GABA transmission in the PVN (Li et al., 2011), it was unexpected that presynaptic GABA density did not correlate with ethanol intake. In contrast, GABA density in AVP axon collaterals correlated with water but not ethanol intake. Again, the construct validity of animal models of ethanol self-administration is of critical importance to interpreting neurobiological mechanisms (Baker et al., 2014). Explants of the PVN show a robust intrinsic inhibitory tone (Bartanusz et al., 2004). *In vivo*, inhibition can be manipulated by microinjection of GABA into the third ventricle, which results in reduced activity of PVN neurons (Plotsky et al., 1987). Importantly, Bartanusz et al. (2004) showed that the intrinsic inhibition within the PVN may modulate, or even override, the glutamatergic signals in this region as glutamate microinjection did not elicit a response unless GABA had been antagonized. While the present data do not show an ethanol-induced change in the relative density of GABA in nerve terminals within the PVN, it is possible that ethanol may be influencing GABA contacts in other ways, perhaps the number of GABA contacts, alterations in postsynaptic function or receptor density, for example. Additionally, the experiments mentioned above as well as the results presented here do not account for changes in noradrenergic signaling within the PVN. Daftary et al. (2000) has shown noradrenergic regulation of parvocellular CRH neurons in the PVN that can be excitatory (via activation of alpha-1 receptors on intranuclear glutamatergic interneurons) or inhibitory via activation of beta-adrenergic receptors. Acute intragastric ethanol administration in rats has been shown to activate brainstem catecholamine regions which regulate the HPA axis via activation of alpha-1 receptors on CRH parvocellular neurons. The effect of long-term ethanol exposure on adrenergic receptors in a primate brain are currently unexplored. The balance of stress hormone receptors is altered postmortem in mood disorder patients in the PVN and prefrontal cortex, involving mineralocorticoid receptors, the receptors for deoxycorticosterone (DOC) and aldosterone (Qi et al., 2013). Unpublished data from our lab suggests that aldosterone response to stress in ethanol-naïve male monkeys predicted future ethanol self-administration. Together, these data argue for additional study of mineralocorticoids and their receptor signaling effects on neurotransmission underlying the stress response, as depression and alcoholism are also comorbid (Sullivan et al., 2005).

In conclusion, the current data provide initial evidence for interactions of ethanol with distinct populations of synaptic terminals in the hypothalamic PVN. As the downstream stress hormones do not show gross disruption following ethanol consumption, appropriate HPA response may be maintained by altered signal integration within the PVN.

## Author Contributions

Dr. KG conceived and designed the experiments. VJ performed the experiments and analyzed the data. VJ and Dr. CH wrote the manuscript. Dr. CM, Dr. AC and Dr. KG edited the manuscript.

## Conflict of Interest Statement

The authors declare that the research was conducted in the absence of any commercial or financial relationships that could be construed as a potential conflict of interest.
